# Single-dose pharmacokinetic and toxicity analysis of pyrrole–imidazole polyamides in mice

**DOI:** 10.1007/s00280-012-1954-3

**Published:** 2012-08-21

**Authors:** Timothy W. Synold, Bixin Xi, Jun Wu, Yun Yen, Benjamin C. Li, Fei Yang, John W. Phillips, Nicholas G. Nickols, Peter B. Dervan

**Affiliations:** 1 Department of Molecular Pharmacology, Beckman Research Institute of the City of Hope, Duarte, CA 91010 USA; 2 City of Hope Medical Center, Duarte, CA 91010 USA; 3 Department of Chemistry and Chemical Engineering, California Institute of Technology, Pasadena, CA 91125 USA

**Keywords:** Py-Im polyamides, Pharmacokinetics, Toxicity, Biodistribution, Mice

## Abstract

**Purpose:**

Pyrrole–imidazole (Py-Im) polyamides are programmable, sequence-specific DNA minor groove–binding ligands. Previous work in cell culture has shown that various polyamides can be used to modulate the transcriptional programs of oncogenic transcription factors. In this study, two hairpin polyamides with demonstrated activity against androgen receptor signaling in cell culture were administered to mice to characterize their pharmacokinetic properties.

**Methods:**

Py-Im polyamides were administered intravenously by tail vein injection. Plasma, urine, and fecal samples were collected over a 24-h period. Liver, kidney, and lung samples were collected postmortem. Concentrations of the administered polyamide in the plasma, excretion, and tissue samples were measured using LC/MS/MS. The biodistribution data were analyzed by both non-compartmental and compartmental pharmacokinetic models. Animal toxicity experiments were also performed by monitoring weight loss after a single subcutaneous (SC) injection of either polyamide.

**Results:**

The biodistribution profiles of both compounds exhibited rapid localization to the liver, kidneys, and lungs upon injection. Plasma distribution of the two compounds showed distinct differences in the rate of clearance, the volume of distribution, and the AUCs. These two compounds also have markedly different toxicities after SC injection in mice.

**Conclusions:**

The variations in pharmacokinetics and toxicity in vivo stem from a minor chemical modification that is also correlated with differing potency in cell culture. The results obtained in this study could provide a structural basis for further improvement of polyamide activity both in cell culture and in animal models.

**Electronic supplementary material:**

The online version of this article (doi:10.1007/s00280-012-1954-3) contains supplementary material, which is available to authorized users.

## Introduction

The development of new DNA-targeted therapeutics is a promising frontier in the treatment of human disease. Pyrrole–imidazole (Py-Im) polyamides are peptides of cyclic aromatic amino acids whose anti-parallel pairing confers sequence-specific binding to the DNA minor groove [1–4]. Members of this class of compounds have been used to modulate gene expression programs in cell culture [5–13] and affect tumor growth in animal models [14–16].

Recently, a series of Py-Im polyamides have been developed to disrupt androgen receptor (AR) signaling [5, 6, 11], presenting an alternative strategy for therapeutic intervention in prostate cancer. These compounds were designed to bind to a 5′-WGWWCW-3′ sequence contained within the consensus androgen response element (ARE) to prevent AR protein–DNA interactions. Cell culture experiments of LNCaP prostate cancer cells co-treated with dihydrotestosterone (DHT) and ARE-targeted polyamides have shown decreased expression of several AR-driven genes such as *PSA, KLK2,* and *TMPRSS2* when compared to samples treated with DHT alone. Polyamide **1** inhibited DHT-induced genes in a dose-dependent manner ranging from 0.74 to 7.4 μg/mL, with 7.4 μg/mL being the most active concentration [11]. Polyamide **2** contains a minor structural modification where the (R)-2,4-diaminobutyric acid turn of **1** is replaced with an acetylated (R)-3,4-diaminobutyric acid. Due to this modification, polyamide **2** was found to have equivalent activity to **1** at tenfold less concentration without significant changes to its DNA-binding ability [6, 8, 11].

While the pharmacokinetics of other Py-Im polyamides have been published previously [17–20], the PK profiles of these structurally distinct ARE-targeted hairpin polyamides have never been explored. For this study, mice were chosen as the preclinical model for the determination of polyamide concentrations in plasma, liver, kidney, and lung. In addition, urinary and fecal levels were measured to assess the relative importance of these routes of drug elimination. The data presented here represent the first detailed description of the in vivo pharmacokinetic and toxicological study of these molecules.

## Materials and methods

### Chemicals and reagents

Acetonitrile (ACN) and methanol (MeOH) were of HPLC grade and purchased from Fisher Scientific (Fair Lawn, NJ, USA). Glacial acetic acid (ACS grade) was purchased from J. T. Baker (Phillipsburg, NJ, USA). Formic acid (99 % pure) was from Acros Organic (New Jersey, USA). Water was purified using the Millipore Milli-Q system (Milford, MA, USA). Mouse plasma for preparation of standards and quality controls (QC) was obtained from The City of Hope Medical Center Animal Center. Py-Im polyamides **1**–**4** were synthesized by solid-phase synthesis as previously described [21, 22]. For structures of internal standards (INS) **3** and **4,** see Online Resource Fig. S1.

### Animals for pharmacokinetic studies

Pyrrole–imidazole polyamide pharmacokinetic studies were performed in 10- to 12-week-old female BALB/C mice (Charles River). Polyamides were solubilized in PBS (**1**) or PBS/DMSO (**2**) and administered via intravenous (IV) tail vein injection at concentrations of 7.5 and 5 mg/kg, respectively. For each experiment, groups of 3 animals were euthanized at 0.083, 0.25, 0.5, 1, 2, 4, 8, and 24 h after injection. Animals designated for the 4, 8, and 24 h time points were housed in metabolic cages for collection of urine and feces as described below. All animals used in the pharmacokinetic experiments were performed under an approved protocol at the City of Hope.

### Animals for toxicology studies

Toxicities of polyamides **1** and **2** were measured after SC injections in 8- to 12-week-old female C57BL/6 mice (Jackson Laboratory). In anticipation of future xenograft experiments, subcutaneous injection, which has been shown to be a viable route of polyamide delivery [20], was chosen as the desired delivery method. A single bolus of polyamide **1** or **2** in PBS/DMSO vehicle was given, and the animals were weighed daily and monitored closely for signs of duress for 7 days. Animals exhibiting >15 % weight loss or signs of distress were euthanized according to regulations outlined by IACUC. Four animals were used in each group unless otherwise noted. This toxicology study was performed under an approved protocol at the California Institute of Technology.

### Analytical methods development

Concentrations of polyamides **1** and **2** were analyzed by LC/MS/MS using a Waters Acquity UPLC system (Milford, MA, USA) interfaced with a Waters Quattro Premier XE Mass Spectrometer. HPLC separation was achieved using a Jupiter 4u Proteo 90A 150 × 2.0 mm column (Phenomenex, Torrance, CA, USA) proceeded by a Phenomenex C_8_ guard column (Torrance, CA, USA). The column temperature was maintained at 30 °C. The mobile phase consisted of A (0.05 % acetic acid in water) and B (0.05 % acetic acid in acetonitrile). The following gradient program was used: 8 % B (0–1 min, 0.3 ml/min), 16 % B (3 min, 0.3 ml/min), 58 % B (6 min, 0.3 ml/min), 90 % B (7 min, 0.3 ml/min), and 8 % B (7.3 min, 0.3 ml/min). The total run time was 11.5 min. The auto-injector temperature was maintained at 5 °C. The strong needle wash solution was 5 % formic acid in MeOH/ACN (2:8) for both compounds, and the weak needle wash solution was 30 % MeOH in water for compound **1** and 50 % ACN in water for compound **2**. The electrospray ionization source of the mass spectrometer was operated in positive ion mode with a cone gas flow of 50 L/hr and a desolvation gas flow of 700 L/hr. The capillary voltage was set to 3.2 kV, and the cone and collision cell voltages were optimized to 32 and 27 V for **1** and the INS **3**. Voltages were optimized to 31 and 20 V for **2** and the INS **4**. The source temperature was 125 °C, and the desolvation temperature was 470 °C. A solvent delay program was used from 0 to 4.0 min and from 6.1 to 11.5 min to minimize the mobile phase to flow to the source. MassLynx version 4.1 software was used for data acquisition and processing.

Positive electrospray ionization of all compounds produced abundant protonated molecular ions (M + 3H) ^3+^. The fragmentations of these compounds were induced under collision-induced dissociation condition. The precursor → product ion combinations at m/z 453.52 → 206.10 for **1**, 454.85 → 210.24 for **3**, 467.45 → 238.32 for **2**, and 469.9 → 238.4 for **4** were used in multiple reaction monitoring (MRM) mode for determination of these compounds. The use of MRM provided sufficient specificity and sensitivity. MS/MS experimental conditions, such as collision energy and collision cell pressure, were optimized from continuous flow injection sample introduction of standard solutions. Under optimized assay conditions, the retention times for **1** and **3** were 5.0 min, and 5.5 min for **2** and **4**.

### Plasma sample preparation

Plasma and urine samples were prepared for LC/MS/MS analysis by mixing 30 μL of plasma with 20 μL of 50 % MeOH and 50 % aqueous 1 % HOAc. The mixture was vortexed and mixed with an additional 120 μL of 0.5 % HOAc in MeOH/ACN (4:6) and 20 μL of 6.0 μg/mL INS in MeOH/1 % aqueous HOAc (1:1). The mixture was vortexed again for 2 min and centrifuged at the highest setting for 4 min. Next, 20 μL of the supernatant was transferred to a new tube and mixed with 180 μL of 50 % MeOH/ACN (4:6) and 50 % aqueous 1 % HOAc.

Standard curves were prepared mixing untreated plasma with 20 μL of 50 % MeOH and 50 % aqueous 1 % HOAc prepared with various concentrations of **1** and **2**. Internal standards were added as described above. The standard curves, as determined by linear regression, displayed good linearity (*r* ^2^ > 0.99) over the range tested for **1** (0.1–30 μg/mL) and **2** (0.2–20 μg/mL).

### Urine and fecal sample preparation

Urine and fecal samples were collected using metabolic cages (Ancare, Techniplast Metabolic Rack, 12 cages by Nalgene). Urine samples were collected at 3 time points over 24 h, and fecal samples were collected at 8 and 24 h time points. Py-Im polyamides were extracted from urine according to the plasma extraction procedure described above.

Fecal samples were first dried at room temperature and then weighed and grounded to a powder. Approximately 100 mg of powder was weighed out and reconstituted in distilled water (6 μL/mg powder). The fecal sample was then homogenized in a TissueLyser (Qiagen) for 2 min at 30 Hz twice, and an additional 6 μL/mg of distilled water was added. Next, 30 μL of the fecal homogenate was mixed with 50 μL distilled water and 20 μL of 50 % MeOH and 50 % aqueous 1 % HOAc. The mixture was then vortex mixed with 0.1 mL 0.5 % HOAc in MeOH/ACN (2:8) and 20 μL of 6.0 μg/mL INS in MeOH/1 % aqueous HOAc (1:1) for 10 min and centrifuged at the highest setting for an additional 10 min. The supernatant was diluted with 50 % MeOH/ACN (4:6) and 50 % aqueous 1 % HOAc.

### Tissue sample preparation

Distribution of polyamides **1** and **2** was determined in the liver, kidneys, and lungs. The organs were harvested post-euthanasia and prepared via similar processes. A piece of the mouse organ was weighed and mixed with distilled water (3 μL/mg tissue). The tissue was then homogenized by pulsing three times on a TissueLyser for 2 min each at 30 Hz. Next, 30 μL of the tissue homogenate was mixed with 20 μL of 50 % MeOH and 50 % aqueous 1 % HOAc. The mixture was then vortex mixed with 0.12 mL 0.5 % HOAc in MeOH/ACN (2:8) and 20 μL of 6.0 μg/mL INS in MeOH/1 % aqueous HOAc (1:1) for 10 min and centrifuged at the highest setting for an additional 10 min. Samples treated with polyamide **1** were then diluted with 50 % MeOH/ACN (4:6) and 50 % aqueous 1 % HOAc. Samples treated with polyamide **2** were diluted with 50 % MeOH/ACN (4:6) and 50 % aqueous 3 % FA.

### Pharmacokinetic data analysis

Plasma pharmacokinetic parameters were derived from polyamide concentration profiles using both non-compartmental and compartmental methods. Non-compartmental analysis was performed according to statistical moment theory and the rule of linear trapezoids, while compartmental analysis was performed in ADAPT II [24]. Pharmacokinetic parameters estimated from the non-compartmental analysis include the maximum concentration (*C*_max_), the terminal elimination half-life (*t*_1/2_), the mean residence time (MRT), the area under the concentration curve (AUC_0–24h_), the AUC extrapolated to infinity (AUC_0–∞_), and the clearance (CL). Additional plasma pharmacokinetic parameters determined from the compartmental analysis include the alpha and beta half-lives (*t*_1/2_) and the apparent volume of distribution (*V*_d_). Tissue pharmacokinetic parameters were determined non-compartmentally and included the *C*_max_ and AUC_0–24h_. Urinary and fecal excretion data were expressed as the cumulative percentage of the administered dose.

### pH stability analysis

The pH stability of Py-Im polyamides was analyzed as previously described [23]. In summary, 15 μL of a 10 μM solution of polyamide **1** or **2** in DMSO was incubated with 85 μL of buffer with pH of 2.5, 4, 7, or 10 (Fluka) at 37 °C for 24 h. After incubation, the sample was mixed with an equal volume of N,N-dimethylformamide and sonicated briefly. Next, 20 μL of the sample solution was mixed with 180 μL of aqueous buffer containing 100 mM NH_4_OAc and 25 μM methyl 4-nitro-1H-pyrrole-2-carboxylate as an internal standard. Analytical HPLC analysis was performed on a Beckman analytical HPLC.

## Results

### Plasma distribution

**Fig. 1:**
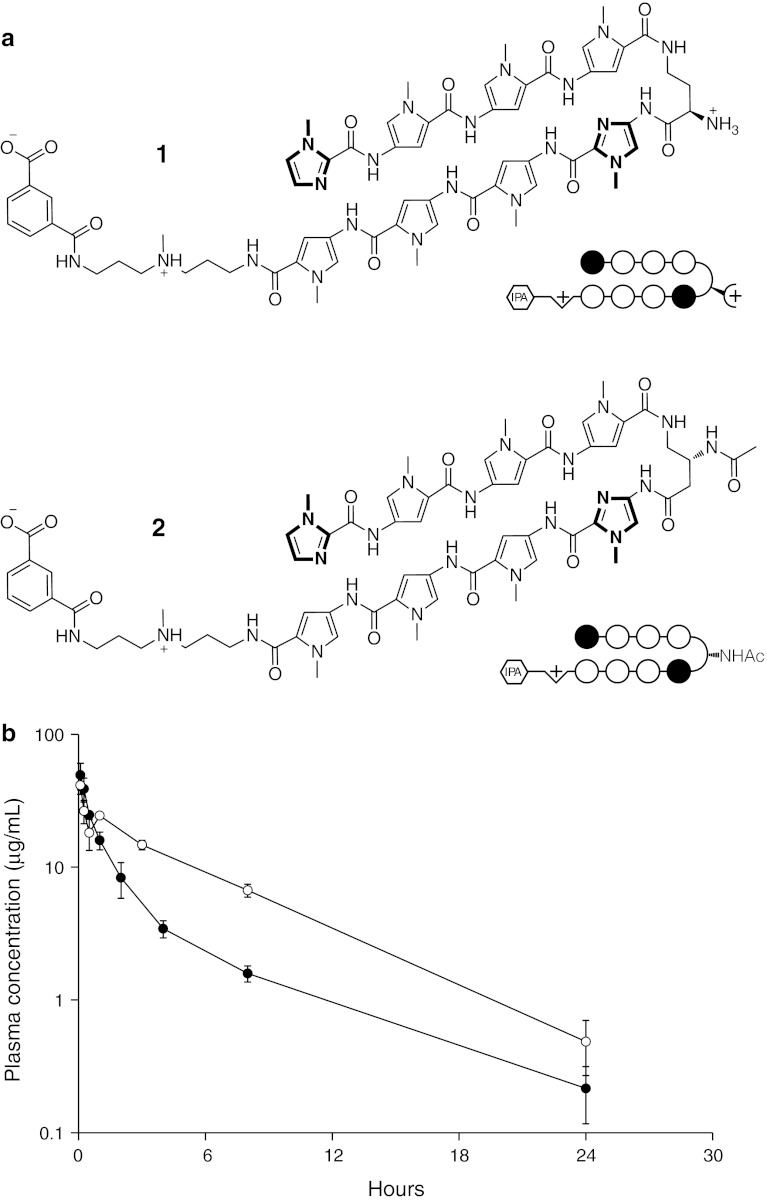
**a** Chemical structures of polyamides **1** and **2**. **b** Plasma concentration versus time curves for **1** (*closed circles*) and **2** (*open circles*). *Error bars* indicate standard deviation

The structures and plasma concentration profiles of polyamides **1** and **2** are shown in Fig. 1, and the pharmacokinetic parameters calculated non-compartmentally and using a 2-compartment model are summarized in Table 1. Plasma concentrations for both polyamides were well above the lower limit of quantification over the entire time course. The average *C*_max_ was 49.4 ± 11.2 μg/mL (mean ± S.D., *n* = 3) for **1** and 41.3 ± 5.9 μg/mL for **2**. Both compounds exhibited a bi-exponential pattern of decay with first-order elimination, with initial and terminal *t*_1/2_’s of 0.5 and 4.6 h for **1**, and 0.1 and 4.2 h for **2**. The average concentrations of **1** and **2** 24 h postinjection were 0.21 ± 0.1 μg/mL and 0.49 ± 0.2 μg/mL, respectively.

**Table 1:** Plasma pharmacokinetic parameters of **1** and **2** after a single IV injection

Parameter	Compound	
	1	2
*I.* *Non*-*compartmental analysis*		
mg/kg	7.5	5
*C*_max_ (μg/mL)	49.4 ± 11.2	41.3 ± 5.9
Elimination *t*_1/2_ (hr)	5.2	4.3
MRT (hr)	3.3	4.9
AUC_0–t_ (μg/mL × hr)	75.8	173.5
AUC_0–∞_ (μg/mL × hr)	77.4	176.5
CL (mL/hr)	1.8	0.6
*II. Compartmental analysis*		
*C*_max_ (μg/mL)	43.6	40.2
*t*_1/2_ α (hr)	0.5	0.1
*t*_1/2_ β (hr)	4.6	4.2
*V*_d_ (mL)	8.1	4
CL (mL/hr)	2.1	0.7
AUC_0–∞_ (μg/mL × hr)	67.5	144.8

Despite using a higher dose, the AUC of **1** was twofold lower than **2** (67.5 vs 144.8 μg/mL × hr). Furthermore, the *V*_d_ of **1** was twofold higher than **2** (8.1 vs 4.0 mL). The calculated CL of **1** was threefold higher than **2** (2.1 vs 0.7 mL/hr).

### Urine and fecal excretion

Concentration profiles of polyamides **1** and **2** in urine are shown in Fig. 3. The urinary excretion of polyamide **1** was nearly complete by 4 h, with a cumulative excretion of 5.7 ± 2.9 % of the administered dose. Urinary excretion of polyamide **2** was much more extensive and continued throughout the entire time course, with a cumulative urinary excretion at 24 h of 46.0 ± 15.2 % of the administered dose.

Fecal recovery at 8 and 24 h did not yield significant amounts of either polyamide, with cumulative recoveries after 24 h of less than 5 % of the administered dose (See Online Resource Fig. S2). This finding is consistent with previously published results of a similarly sized polyamide [19].

### Tissue distribution

To examine tissue distribution, several organs previously reported to have polyamide localization were analyzed. Distribution profiles of polyamides **1** and **2** in the liver are shown in Fig. 3a. Both compounds localized rapidly to the liver post-administration. Polyamide **1** reached a maximum concentration of 11.7 ± 1.3 μg/g at 5 min postinjection. The concentration of polyamide **2** also peaked 5 min after injection at a maximum concentration of 43.8 ± 0.7 μg/g. Both polyamides exhibited higher retention in the liver tissue than plasma. At the experiment endpoint, 4.8 ± 0.3 μg/g of **1** and 17.4 ± 8.1 μg/g of **2** were found to remain in the liver. The AUC_0–24h_ of polyamides **1** and **2** in the liver were 157.7 and 301.3 μg/g × hr, respectively. The localization of **1** and **2** to the liver is consistent with previously published positron emission tomography (PET) results of a related radiolabeled hairpin polyamide [23].

Polyamide pharmacokinetic profile in the kidneys is shown in Fig. 3b. Maximum kidney concentration of both polyamides was reached 5 min postinjection with an average *C*_max_ of 27.0 ± 2.9 μg/g and 35.1 ± 2.8 μg/g for polyamides **1** and **2**, respectively. As in liver, the rate of polyamide elimination from the kidney was slower than from the plasma, and the AUC_0–24h_ of polyamides **1** and **2** in the kidney was 299.2 and 424.7 μg/g × hr, respectively. The increased concentrations of polyamide **2** relative to **1** in kidney were consistent with its higher rate of urinary excretion.

Unlike liver and kidney, polyamide concentrations in the lung peaked at 15 min following injection for both compounds (Fig. 3c). The *C*_max_ of polyamide **2** in the lung was greater than 15-fold higher than compound **1**, with maximum concentrations of 256 ± 93.1 μg/g for **2** and 16.4 ± 1.4 μg/g for **1**. After an initial rapid decline, especially for polyamide **2**, concentrations in the lung were maintained above 2.8 ± 0.2 μg/g and 21.8 ± 7.6 μg/g for **1** and **2,** respectively over the entire time course. The AUC_0–24h_ of polyamides **1** and **2** in the lung was 130.6 and 523.5 μg/g × hr, respectively. Tissue PK parameters are summarized in Table 2.

**Table 2:** Tissue pharmacokinetic parameters of **1** and **2** after a single IV injection

Compound	**1**		**2**	
	*C*_max_ (μg/g)	AUC (μg/g × hr)	*C*_max_ (μg/g)	AUC (μg/g × hr)
Liver	11.7 ± 1.3	157.7	43.8 ± 0.7	301.3
Kidney	27.0 ± 2.9	299.2	35.1 ± 2.8	424.7
Lung	16.4 ± 1.4	130.6	256 ± 93.1	523.5

### Compound stability

The stability of polyamides **1** and **2** at various physiological pHs was explored by incubating in pH 2.5, 4, 7, and 10 buffers at 37 °C for 24 h. Analytical HPLC analysis of incubated samples did not display significant signs of degradation at any pH. See Online Resource Fig. S3.

### Toxicity study

Based on a defined threshold of greater than 15 % weight loss over a 7-day observation period, the toxicity following a single subcutaneous injection of polyamide **1** or **2** was determined to be significantly different (Fig. 4). For polyamide **1**, critical weight loss occurred only at the highest dose level 10 mg/kg. However, polyamide **2** demonstrated dose-limiting weight loss at both 4.5 and 2.3 mg/kg. No additional signs of duress were observed in the animals treated with polyamide **1**; however, animals treated with polyamide **2** at doses of 4.5 and 2.3 mg/kg exhibited multiple signs of duress such as loss of ambulation and hunched posture in addition to weight loss.

## Discussion

Pyrrole–imidazole polyamides are sequence-specific DNA minor groove binders that have been shown to modulate gene expression regulated by transcription factors of oncological importance [10–13]. Of these compounds, two hairpin polyamides developed to disrupt AR signaling are of particular interest due to their gene regulation activities [6, 11] and potent cytotoxicity toward the LNCaP prostate cancer cell line [25]. While the two hairpin polyamides are structurally similar, a minor structural modification on the diaminobutyric acid turn was able to confer a tenfold increase in the ability of polyamide **2** to downregulate *PSA* mRNA expression. In this study, pharmacokinetic methods were employed to explore the differences in circulation, excretion, and tissue biodistribution of these ARE-targeted hairpin polyamides in mice.

**Fig. 2:**
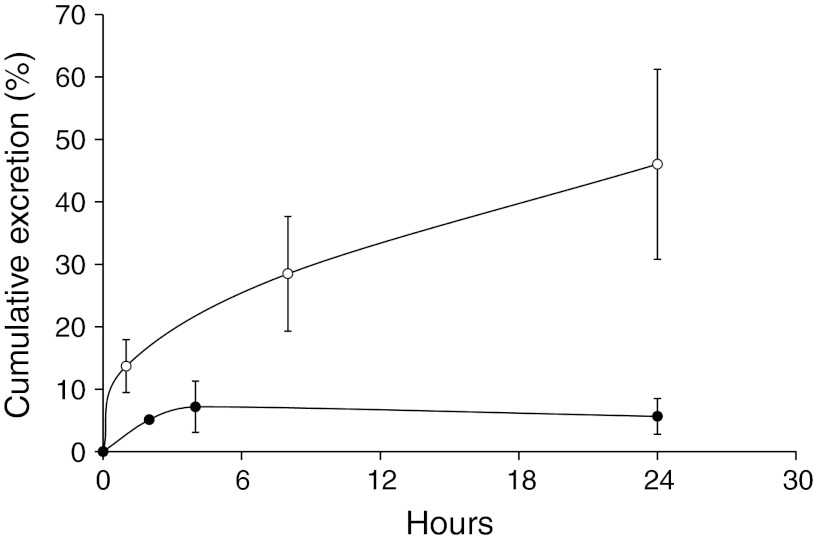
Cumulative percent urinary excretion of **1** (*closed circles*) and **2** (*open circles*). *Error bars* indicate standard deviation

Polyamide distribution in the plasma showed clearance profiles indicative of first-order elimination for both compounds (Fig. 1b; Table 1). These data are in line with published PK results of related polyamides in rats [17]. The maximum plasma concentration for polyamide **1** was found to be over 3 times the effect dosage for *PSA* mRNA downregulation in cell culture, while the *C*_max_ for polyamide **2** was found to be approximately 29 times the effective concentration. Analysis of the plasma PK data showed that polyamide **2** exhibited a higher systemic exposure and lower clearance rate than polyamide **1**. Although the plasma clearance of polyamide **1** was ~threefold faster than polyamide **2,** it was not significantly eliminated through the urine or feces. Polyamide **2**, however, was largely eliminated through the urine (Fig. 2). The low amount of renal and biliary elimination of compound **1** may be suggestive of compound retention in the tissues or its metabolic degradation. A previous absorption, distribution, metabolism, excretion, and toxicity (ADMET) study had ascertained that polyamide **2** was resistant to liver microsomal degradation [5]; however, the microsomal stability of polyamide **1** was never examined, and thus, enzymatic degradation could be a route of elimination for this compound.

**Fig. 3:**
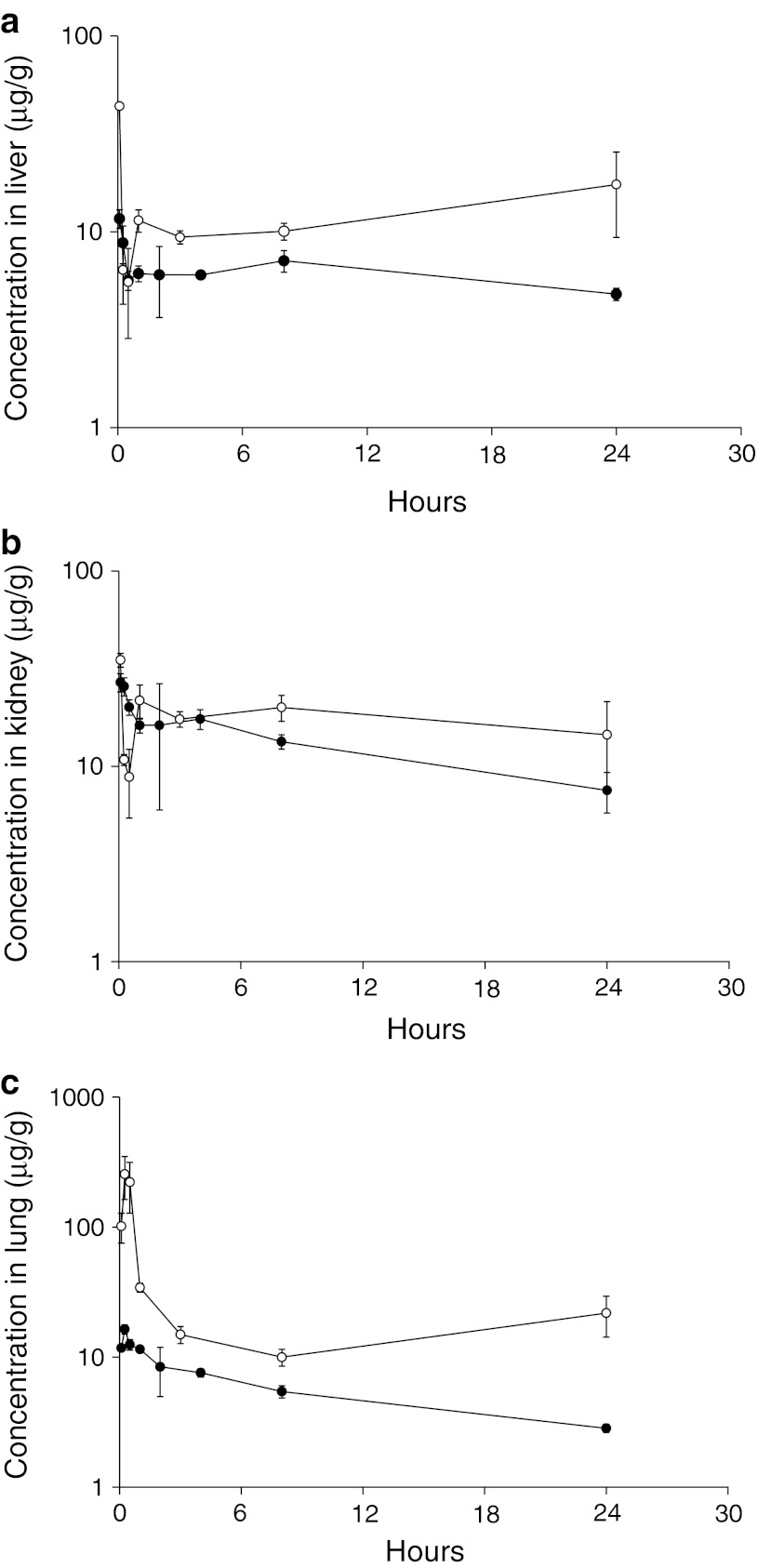
Concentration versus time curves in **a** liver, **b** kidney, and **c** lung for **1** (*closed circles*) and **2** (*open circles*). *Error bars* indicate standard deviation

Interestingly, tissue analysis of the liver, kidneys, and lungs showed higher concentrations of polyamide **2** than **1** (Fig. 3; Table 2). The three organs analyzed here have been previously documented as representative organs of polyamide localization [17, 23]; however, it is likely that the compounds were also taken up and retained in other tissues types, and that similar differences between the polyamides may exist in these sites. The differences in biodistribution between the two compounds may be attributable to differences in solubility. Polyamide **2** is less soluble than **1** in aqueous solutions and requires a polar aprotic cosolvent like DMSO for administration. Because the initial distribution of polyamide **2** to lung tissue was more than 15-fold higher than polyamide **1,** it is possible that compound **2** is precipitating out of solution as it reaches high local concentrations when passing through the lung immediately after an intravenous injection. Alternatively, it is possible that polyamide **2** is preferentially taken up and retained by the lung tissue itself. This phenomenon has been previously described for many drugs and exogenous compounds, and the lungs have been demonstrated to have significant effects on the pharmacokinetics of drugs given intravenously [26]. Regardless of the mechanism of accumulation, once the concentration of polyamide **2** peaks in the lung, it apparently redistributes unchanged back into circulation as indicated by a second peak in the plasma concentration versus time profile. Therefore, rather than being a site for drug elimination, the lung is serving as a reservoir for polyamide **2** and merely delays its release back into the central compartment.

**Fig. 4:**
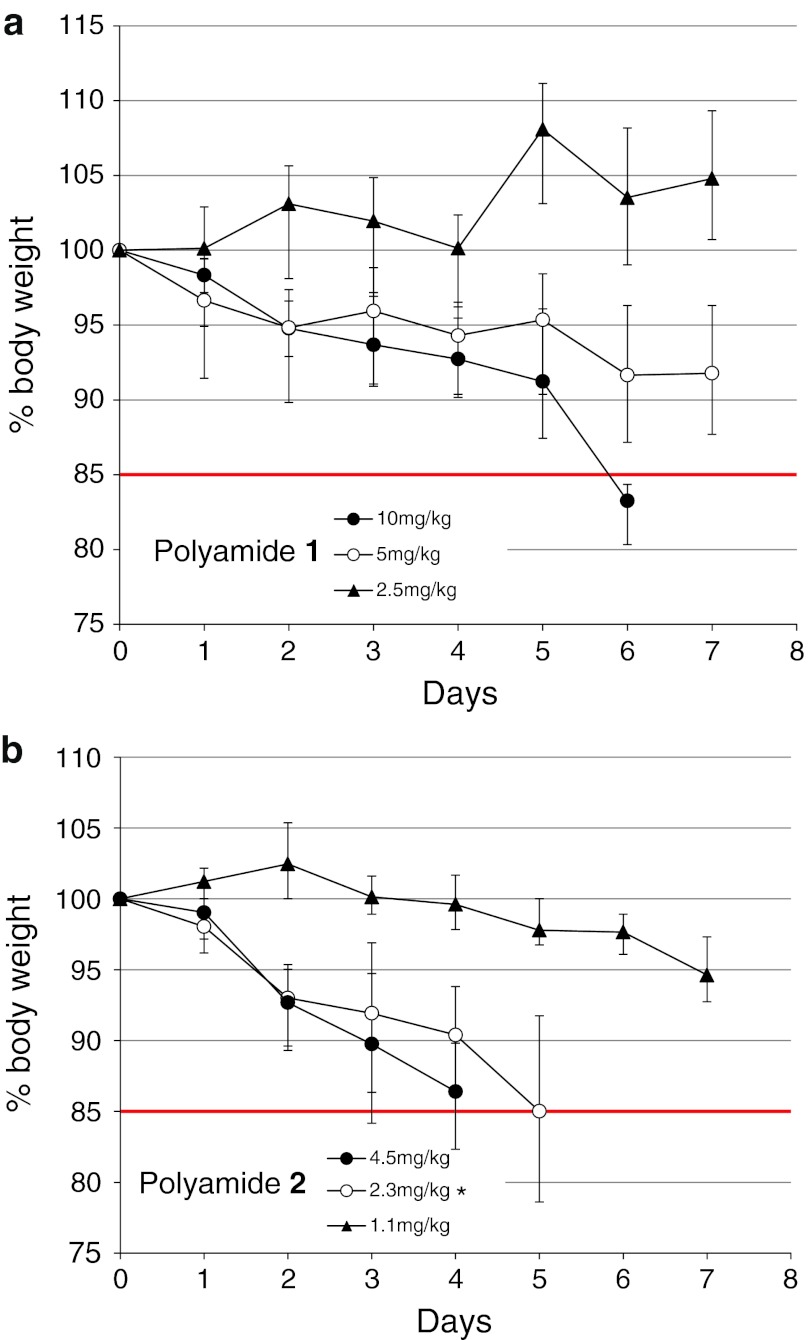
Animal toxicity experiments for polyamides: **a,** **1** and **b,** **2**. Animals were injected on day 0 and monitored for 7 days for weight loss and signs of duress. *Error bars* indicate standard deviation, *n* = 4 (*n = 3)

In addition to differences in biodistribution, animal toxicity studies also revealed major differences between the two compounds. Weight curve experiments following a single SC injection of **1** and **2** showed polyamide **2** to be more toxic (Fig. 4). Animals treated with **1** only showed significant weight loss at a dose of 10 mg/kg, and no additional sign of duress was observed. In contrast, animals treated with polyamide **2** exhibited additional signs of physical duress in addition to weight loss at all concentrations except 1.1 mg/kg. Taken together, given its greater potency against the expression of select AR-driven genes and its higher accumulation in normal tissues, the increased toxicity of polyamide **2** is likely due to off-target effects in normal organs. However, an alternative explanation for the increased toxicity seen with polyamide **2** could also be due to its relatively poor aqueous solubility. For example, in tissues where high local concentrations of polyamide **2** are achieved (i.e., lung), the compound may precipitate in capillaries, resulting in microinfarctions and ischemic tissue injury.

In conclusion, both polyamides **1** and **2** are bioavailable in mice after IV tail vein injection, and plasma concentration of both compounds is well above the levels required for gene regulation in cell culture. Although polyamide **2** exhibited more favorable plasma PK characteristics, with a higher AUC and slower clearance from plasma, it was found to be significantly more toxic to the animals. This study was the first to explore the PK properties of ARE-targeted hairpin polyamides, and it has revealed how a minor structural modification can influence the PK and toxicological properties of polyamides, thus setting the ground work for future xenograft experiments and providing a potential route to improve polyamide design for clinical applications.

## Electronic supplementary material

Below is the link to the electronic supplementary material.

Supplementary material 1 (DOCX 147 kb)
