# Supplementary material for: Single-dose pharmacokinetic and toxicity analysis of pyrrole–imidazole polyamides in mice
Source: Cancer Chemother Pharmacol. 2012 Aug 21;70(4):617–25. doi: 10.1007/s00280-012-1954-3 (PMC3456924; doi:10.1007/s00280-012-1954-3)

Supplemental Information

Single-dose pharmacokinetic and toxicity analysis of pyrrole-imidazole polyamides in mice


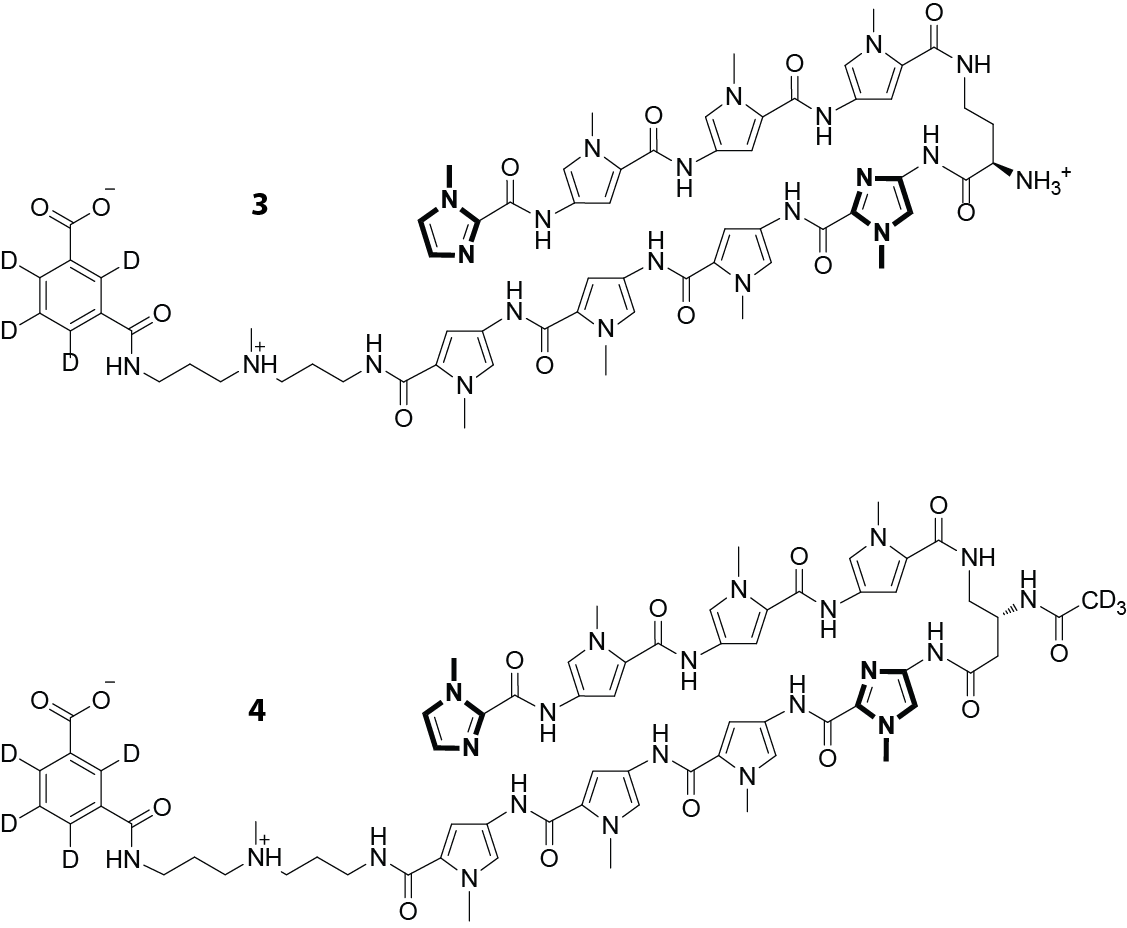
**Fig S1** Chemical structures of standards **3** and **4** used in LC/MS/MS analysis. Polyamide **3** was used as the internal standard for **1**, and polyamide **4** was used as the standard for **2**

**Fig S2** Fecal concentrations of polyamides **(a)** **1** and **(b)** **2** as percentage of original intravenous dose.

Open circles represent individual measurements and the average is represented by a solid line


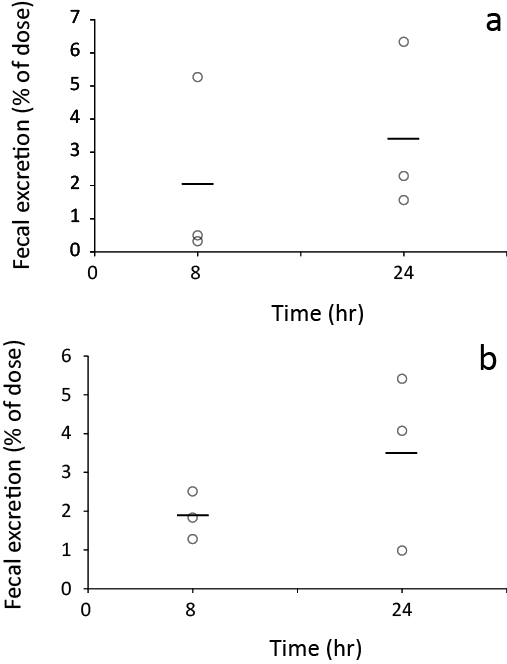


**Fig S3** Stability of compounds **(a)** **1** and **(b)** **2** when incubated at the indicated pHs at 37^o^C for 24hr. **(c)** Standard only


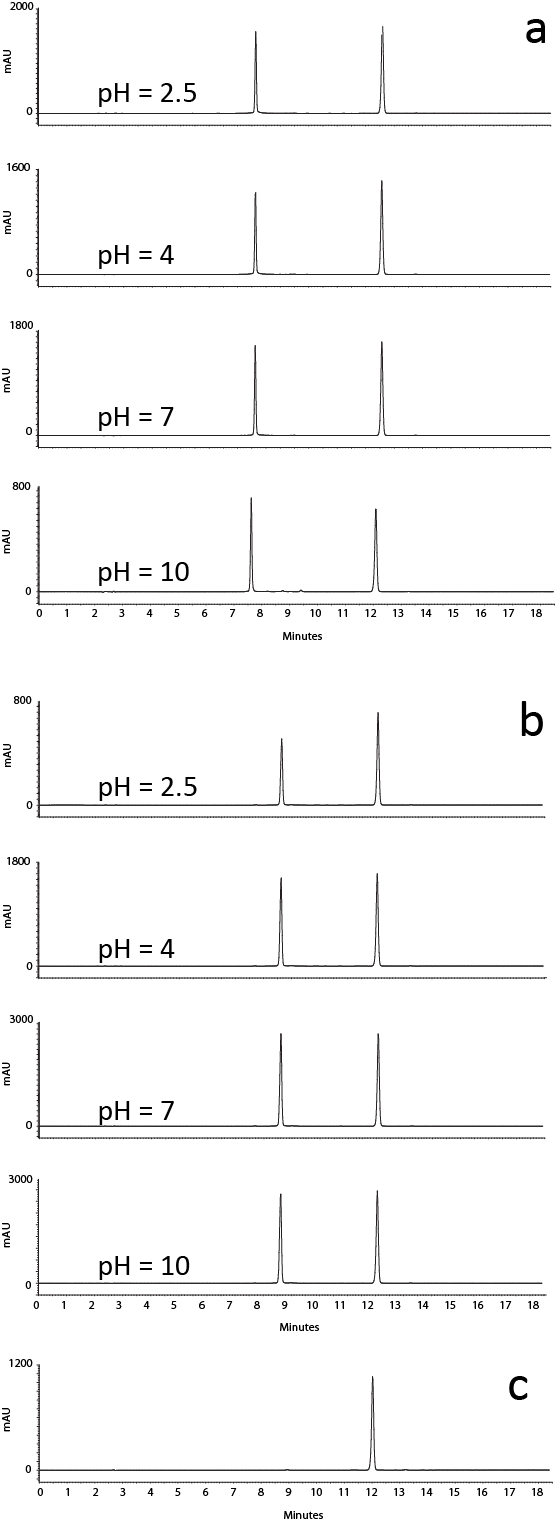

Supplement: Supplementary file 1 — Supplementary material 1 (DOCX 147 kb) [file 280_2012_1954_MOESM1_ESM.docx]
